# Carotid Plaque Characteristics Combined With Serum Inflammatory Biomarkers Predict Recurrent Ischemic Stroke

**DOI:** 10.1002/brb3.70909

**Published:** 2025-10-07

**Authors:** Lin Wang, Tong Chen, Chun Yuan, Thomas Hatsukami, Xihai Zhao, Mi Shen, Peiyi Gao, Xingquan Zhao, Binbin Sui

**Affiliations:** ^1^ Department of Neurology Beijing Tiantan Hospital Capital Medical University Beijing China; ^2^ Department of Neurology Beijing Daxing Hospital Capital Medical University Beijing China; ^3^ Department of Radiology Beijing Tiantan Hospital Capital Medical University Beijing China; ^4^ Tiantan Neuroimaging Center for Excellence China National Clinical Research Center for Neurological Diseases Beijing Neurosurgical Institute Beijing Tiantan Hospital Beijing China; ^5^ Center for Biomedical Imaging Research Department of Biomedical Engineering Tsinghua University School of Medicine Beijing China; ^6^ Department of Radiology and Imaging Sciences, Spencer Fox Eccles School of Medicine University of Utah Salt Lake City Utah USA; ^7^ Department of Surgery University of Washington Seattle Washington USA; ^8^ China National Clinical Research Center for Neurological Diseases Capital Medical University Beijing China; ^9^ Research Unit of Artificial Intelligence in Cerebrovascular Disease Chinese Academy of Medical Sciences Beijing China; ^10^ Department of Radiology, Beijing Hospital. National Center of Gerontology, Institute of Geriatric Medicine, Chinese Academy of Medical Sciences. China

**Keywords:** carotid atherosclerotic, high‐resolution vessel wall imaging, inflammatory biomarkers, recurrent stroke

## Abstract

**Objective:**

To investigate the potential value of serum inflammatory markers combined with carotid plaque characteristics for predicting subsequent recurrent ischemic events.

**Methods:**

Patients with cerebral infarction localized to the internal carotid artery territory were recruited. Carotid MRI examinations were performed, and the plaque characteristics were evaluated at baseline. Serum samples were obtained, and clinical characteristics were documented at baseline. All participants received clinical follow‐up 1 year after discharge. Recurrent ipsilateral ischemic stroke was considered the clinical endpoint. Logistic regression analyses were employed to assess the correlations between serum inflammatory biomarkers, plaque characteristics, and the endpoint. The diagnostic performances were evaluated using receiver operating characteristic curves. DeLong's test was used to compare the areas under the curve (AUCs) of the models.

**Results:**

In total, 89 patients (84.3% men; mean age, 56.57 ± 9.05 years) with recent anterior circulation (carotid territory) cerebral hemisphere ischemia were included. Sixteen patients presented with an endpoint within the 1‐year follow‐up. Multivariate logistic regression demonstrated that the normalized wall index, intraplaque hemorrhage, lipid‐rich necrotic core, and high‐sensitivity C‐reactive protein (Hs‐CRP) were associated with the endpoint. The model combining plaque characteristics and Hs‐CRP had the highest diagnostic performance with an AUC of 0.855.

**Conclusions:**

Serum Hs‐CRP level and plaque characteristics may provide complementary information for predicting recurrent stroke. The combination of these two indicators may be a better potential predictor of the population at high risk of stroke recurrence.

## Graphical abstract

This study explored the value of combining serum inflammatory markers with carotid high‐resolution vessel wall imaging in predicting recurrent stroke. Our findings indicated that the combination of serum Hs‐CRP levels and plaque characteristics may provide complementary information for predicting the population at high risk of stroke recurrence.

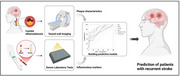

**Abbreviations**
AUC: area under the curve
HR‐VWI: high‐resolution vessel wall imaging
Hs‐CRP: high‐sensitivity C‐reactive protein
IPH: intraplaque hemorrhage
IQR: Interquartile range
LRNC: lipid‐rich necrotic core
NLR: neutrophil‐to‐lymphocyte ratio
NWI: normalized wall index
ROC: Receiver operating characteristic curve
SD: Standard deviation
TIA: Transient ischemic attack

## 1 Introduction

Stroke is one of the primary causes of death globally and is also characterized by high morbidity, disability, and mortality, especially in China (Lozano et al. 2012; W. Wang et al. 2017; GBD 2019 Diseases and Injuries Collaborators 2020). Although the stroke recurrence rate has markedly declined in China, approximately 12.5% of patients still experience recurrent stroke within 12 months (Han et al. 2024). Carotid atherosclerotic disease has been found in around 15%–20% of patients with ischemic cerebrovascular events (Cheng et al. 2019). Epidemiological research indicates that carotid atherosclerosis is increasingly recognized as a primary contributor to ischemic stroke (Lozano et al. 2012).

Three‐dimensional high‐resolution vessel wall imaging (HR‐VWI) represents an innovative approach for quantitative evaluation of plaque morphology and composition (Mandell et al. 2017). Carotid HR‐VWI enables measuring arterial wall structural parameters, and multi‐contrast weighted scanning allows evaluating atherosclerotic compositional features (Pakizer et al. 2025). Vulnerable plaque components, including the lipid‐rich necrotic core (LRNC) and intraplaque hemorrhage (IPH), have been correlated with recurrent stroke (Kwee et al. 2013). A recent study suggests that plaque volume and IPH serve as independent risk factors for recurrent ischemic events in individuals with mild‐to‐moderate carotid stenosis (van Dam‐Nolen et al. 2022).

Inflammation is considered to play a crucial role in the progression of atherosclerosis and thrombosis (Martinez et al. 2020). Much discussion has taken place regarding serum inflammatory markers associated with atherosclerosis. A review of serum biomarkers in carotid atherosclerosis suggested that lipids, homocysteine (HCY), and interleukins can be used to assess the risk correlated with carotid atherosclerosis (Martinez et al. 2020). Several studies showed that higher inflammatory marker levels were related to higher recurrent stroke rates (Li et al. 2021, 2016; Ojima et al. 2020).

Carotid plaque characteristics and serum inflammatory marker levels are important factors for determining stroke recurrence; however, whether the combination of the two indicators can help better predict stroke recurrence remains uncertain. Therefore, this study aimed to explore the value of serum inflammatory markers combined with carotid plaque characteristics on HR‐VWI in predicting subsequent recurrent ischemic events.

## 2 Methods

### 2.1 Participants

This study was a post hoc analysis of a prospective observational study. Patients were prospectively recruited at Beijing Tiantan Hospital. Patient inclusion criteria were (1) aged 30–79 years; (2) presented with anterior circulation (carotid territory) cerebral hemisphere ischemia; (3) had detailed baseline clinical information. Patient exclusion criteria were (1) life expectancy < 1 year; (2) suspected cardiogenic embolism or cerebral infarction secondary to cerebral hemorrhage, trauma, tumor, abnormal clotting mechanism, aneurysm or arteriovenous malformation or unknown etiology; (3) receipt of thrombolysis, emergency interventional therapy, or stenting in symptomatic arteries; (4) non‐cerebrovascular disease with focal or global brain symptoms or unclear diagnosis of cerebrovascular disease; (5) severe inflammatory diseases (rheumatoid arthritis, infections, osteoarthritis, and/or cirrhosis; (6) recent trauma, severe heart failure, surgery, liver or kidney insufficiency, autoimmune disease, hematologic disease, and/or peripheral vascular disease; (7) inability to undergo magnetic resonance imaging (MRI); and (8) severe stenosis (> 50%) of the ipsilateral intracranial artery. The bilateral carotid 3D HR‐VWI scans were performed within 7 ± 3 days of admission. Arteries ipsilateral to the ischemic symptoms were considered symptomatic following a published method (Zhao et al. 2017). All patients had provided written informed consent before the study. This study was reviewed and approved by the Ethics Committee of Beijing Tiantan Hospital (KY2014‐004‐04).

### 2.2 Clinical Assessment

Demographic and clinical information was obtained from the medical records. Hypertension, hyperlipidemia, and diabetes mellitus were determined and recorded (Che et al. 2022). Coronary heart disease (a condition caused by atherosclerosis that typically results in angina pectoris or myocardial infarction) and a family history of stroke (a history of stroke in at least one first‐degree relative of the patient) were recorded, as were smoking history (past or current), aspirin use, and statin use.

### 2.3 Carotid HR‐VWI Protocol

All examinations were performed on a 3T MRI scanner (Trio‐Tim, Siemens Healthcare, Erlangen, Germany) equipped with an eight‐channel head and carotid surface coil (TSImaging Healthcare, Beijing, China). The carotid HR‐VWI protocol included 3D time‐of‐flight magnetic resonance angiography (TOF‐MRA), 3D magnetization‐prepared rapid acquisition gradient‐echo (3D MP‐RAGE), and pre‐ and post‐contrast VWI sequences. Table S1 lists the detailed parameters of these imaging sequences.

### 2.4 MRI Analysis

The Vesselexplorer2 software (TSImaging Healthcare) was used to post‐process the HR‐VWI images. Two neuroradiologists experienced in HR‐VWI analysis reviewed all carotid images, and they were blinded to the patients' information. The image quality was evaluated according to previously published four‐point criteria (Zhou et al. 2015), and poor image quality (image quality = 1) was excluded. Conflicts between the reviewers were settled through consultation.

At each axial section of the carotid arteries, the boundaries of the lumen, outer wall, and each plaque component were manually outlined. The plaque characteristics were recorded and calculated. The normalized wall index (NWI) (NWI = wall area/(lumen area + wall area) × 100%) was calculated. The degree of luminal stenosis was determined on the TOF‐MRA images with a maximum intensity projection algorithm using NASCET criteria (Ferguson et al. 1999).

The plaque compositions were identified using the following criteria. IPH was determined when a hyperintensity > 150% of the muscle on the TOF‐MRA, T1‐SPACE, or MP‐RAGE images. Calcification was characterized by a hypointense appearance on all sequences. The LRNC was isointense both on the TOF‐MRA and T1‐SPACE and hypointense on the post‐contrast T1‐SPACE images within the plaque.

### 2.5 Laboratory Analysis

On admission to the emergency department or hospital ward, all laboratory tests were performed using blood collected from the anterior cubital vein after a 12‐h fast. Serum levels of the routine blood parameters, lipids, Hs‐CRP, and HCY were measured using standard test methods. The neutrophil‐to‐lymphocyte ratio (NLR) (the neutrophil count/the lymphocyte count) was also calculated.

### 2.6 Clinical Follow‐Up of Recurrent Stroke

All subjects were followed up by telephone interview (Che et al. 2022; Li et al. 2020) 1 year after discharge. Ipsilateral recurrent stroke was defined as a sudden onset of focal neurological deficit lasting more than 24 h within the ipsilateral carotid arterial territory after the initial stroke (Marnane et al. 2014; Lu et al. 2018). Two experienced stroke neurologists diagnosed recurrent stroke based on the clinical symptoms described by the patients or their agents, as well as their complete imaging examinations. Patients with missing information (including patients who could not be reached by telephone) and patients who died without hospitalization were excluded.

### 2.7 Statistical Analysis

Categorical variables are reported as numbers (%). Depending on the distribution of the data, continuous variables are reported as mean ± standard deviation (SD) or medians (interquartile range [IQR]). Differences between groups for categorical variables were evaluated by the chi‐squared test. The Mann–Whitney *U* test or Student's *t*‐test was performed to analyze differences in continuous variables. Taking recurrent stroke as the dependent variable, the correlation between the variables and recurrent ischemic events was analyzed using logistic regression analyses. Receiver operating characteristic (ROC) curves and areas under the curve (AUCs) were calculate to evaluate the models’ diagnostic performances. The AUCs of the different models were compared by DeLong's test (DeLong et al. 1988). *p *< 0.05 was considered statistically significant. All statistical analyses were conducted with SPSS software 25.0 (IBM, Armonk, NY, USA).

## 3 Results

### 3.1 Demographic and Clinical Characteristics

**FIGURE 1:**
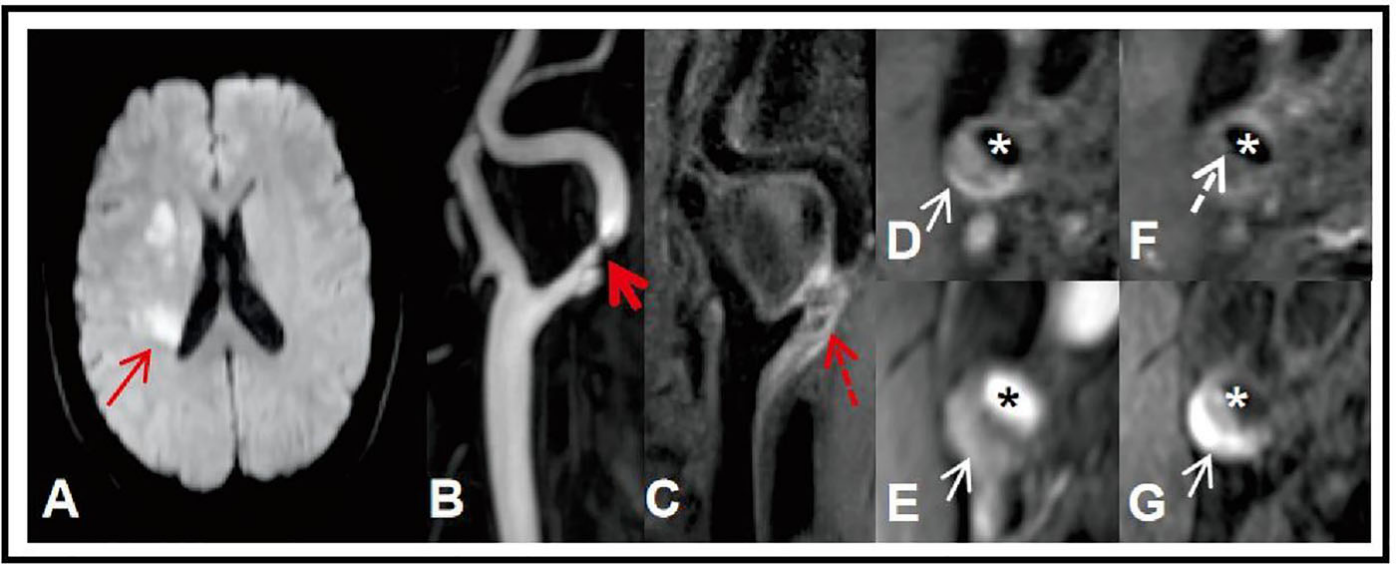
A representative case with stroke recurrence. A 59‐year‐old man with an ischemic stroke. The Serum Hs‐CRP level was 10.4 mg/L. He had an ipsilateral recurrence 10 months after discharge. DWI (A) showed a patchy hyperintensity lesion at the right corona radiata area (thin red arrow). CE‐MRA (B) showed severe stenosis at the right ICA (thick red arrow). Pre‐contrast T1W reformat image (C) displayed a large plaque involving the carotid bifurcation and the beginning of the ICA (red dotted arrow). NWI was 0.94. The plaque showed heterogeneous signal intensity, with a large area of hyperintensity on Pre‐contrast axial T1W (D), 3D TOF MRA (E), and 3D MP‐RAGE (G), indicating IPH (white arrow). Post‐contrast T1W (F) images showed a large non‐contrast area, suggesting LRNC with a thin/ruptured FC (white dotted arrow). 3D MP‐RAGE, 3D magnetization‐prepared rapid acquisition gradient‐echo; 3D TOF, 3D time‐of‐flight; CE‐MRA, Contrast‐enhanced magnetic resonance angiography; DWI, diffusion‐weighted imaging; FC, fibrous Cap; Hs‐CRP, high‐sensitivity C‐reactive protein; ICA, internal carotid artery; IPH, intraplaque hemorrhage; T1W, T1‐weighted.

From June 2015 to December 2018, a total of 110 patients completed carotid HR‐VWI. Nine patients were excluded for poor image quality, three patients were excluded because they lacked 24‐h blood samples, four patients were excluded because they had emergency or interventional thrombolysis, and five were lost to follow‐up. Finally, 89 patients (75 men [84.3%]; mean age, 56.57 ± 9.05 years) were included to be analyzed. Figure S1 shows the patient inclusion flowchart. Sixteen of these (17.9%) presented with recurrent stroke (Figure 1); 26 (29.2%) presented with plaque calcification; 13 (14.6%) presented with plaque with LRNC, and 11 (12.4%) presented with IPH.

Age, sex, hypertension, diabetes mellitus, body mass index, hyperlipidemia, coronary heart disease, prior ischemic cerebrovascular events, family history of stroke, and medication history didn't show significant differences between patients with and without stroke recurrence (all *p *> 0.05). Patients with recurrent stroke showed higher Hs‐CRP levels (*p *< 0.001), higher NLR (*p *= 0.034), and lower high‐density lipoprotein cholesterol levels (*p *= 0.018) compared to those without recurrent stroke. The detailed demographic and clinical characteristics are displayed in Table 1.

**TABLE 1:** Patient demographics and clinical characteristics.

Variable	Overall (*n* = 89)	Ischemic stroke recurrence within 1 year		*p* value
		No (*n* = 73)	Yes (*n* = 16)	
Sex, male, *n* (%)	75 (84.3)	63 (85.1)	12 (80.0)	0.618
Age, years	56.5 ± 9.0	56.3 ± 9.2	57.6 ± 8.1	0.632
BMI, kg/m^2^	24.7 ± 3.2	24.8 ± 3.2	24.1 ± 3.2	0.457
Hypertension, *n* (%)	56 (62.9)	48 (64.9)	8 (53.3)	0.399
Diabetes mellitus, *n* (%)	36 (40.4)	31 (41.9)	5 (33.3)	0.538
Coronary heart disease, *n* (%)	8 (9.0)	7 (9.5)	1 (6.7)	0.730
Hyperlipidemia, *n* (%)	17 (19.1)	13 (17.6)	4 (26.7)	0.414
Prior stroke/TIA, *n* (%)	19 (21.3)	15 (20.3)	4 (26.7)	0.581
Family history of stroke, *n* (%)	31 (34.8)	28 (37.8)	3 (20.0)	0.186
Smoking history, *n* (%)	59 (66.3)	49 (66.2)	10 (66.7)	0.973
Alcohol consumption, *n* (%)	48 (53.9)	40 (54.1)	8 (53.3)	0.959
Hs‐CRP, IQR, mg/L	1.5 (0.8, 2.9)	1.3 (0.6, 2.4)	5.7 (2.7, 11.8)	<0.001
NLR	2.5 (1.8, 3.6)	2.4 (1.8, 3.3)	3.2 (2.2, 4.8)	0.034
HCY, IQR, µmol/L	12.2 (10.1, 16.9)	11.8 (9.9, 16.4)	14.3 (11.8, 29.8)	0.069
Triglycerides (mmol/L)	1.3 (0.9, 1.8)	1.3 (0.9, 1.8)	1.0 (0.9, 1.7)	0.431
Total cholesterol (mmol/L)	3.7 ± 0.9	3.7 ± 0.9	3.4 ± 0.7	0.327
High‐density lipoprotein cholesterol (mmol/L)	0.9 (0.8, 1.1)	0.9 (0.8, 1.1)	0.8 (0.7, 1.0)	0.018
Low‐density lipoprotein cholesterol (mmol/L)	2.0 (1.6, 2.6)	2.0 (1.6, 2.7)	2 (1.7, 2.5)	0.951
Aspirin use until primary outcome, *n* (%)	52 (58.4)	43 (58.1)	9 (60.0)	0.892
Statins use until primary outcome, *n* (%)	45 (50.6)	38 (51.4)	7 (46.7)	0.741

Abbreviations: BMI, body mass index; HCY, homocysteine; Hs‐CRP, high‐sensitivity C‐reactive protein; NLR, neutrophil‐to‐lymphocyte ratio; TIA, Transient ischemic attack.

### 3.2 Carotid Plaque Characteristics in HR‐VWI

Patients with recurrent stroke showed a significantly higher NWI (*p *= 0.008), higher LRNC presence, and more IPH (both *p *< 0.001) than those without recurrent stroke. No significant difference was found in the degree of stenosis (*p *= 0.147). The detailed carotid plaque characteristics are displayed in Table 2.

**TABLE 2:** Carotid atherosclerotic plaque characteristics on HR‐VWI.

Variable	Overall (*n* = 89)	Ischemic stroke recurrence within 1 year		*p* value
		No (*n* = 73)	Yes (*n* = 16)	
Category of stenotic degree				0.147
< 30%, *n* (%)	23 (25.8)	17 (23.0)	6 (40.0)	
30%–49%, *n* (%)	54 (60.7)	45 (60.8)	9 (60.0)	
> 50%, *n* (%)	12 (13.5)	12 (16.2)	0 (0)	
NWI, %	60 (60, 70)	60 (60, 70)	70 (60, 90)	0.008
Presence of plaque components, *n* (%)				
Calcification	26 (29.2)	21 (28.4)	5 (33.3)	0.700
LRNC	13 (14.6)	6 (8.1)	7 (46.7)	< 0.001
IPH	11 (12.4)	5 (6.8)	6 (40.0)	< 0.001

Abbreviations: IPH, intraplaque hemorrhage; LRNC, lipid‐rich necrotic core; NWI, normalized wall index.

### 3.3 Logistic Regression Analyses for Recurrent Stroke

Univariate logistic regression analysis found that Hs‐CRP (odds ratio [OR] = 1.37, 95% confidence interval [CI] 1.16–1.61, *p *< 0.001), HDL (OR = 0.03, 95% CI, 0.00–0.76, *p = *0.033), NWI (OR = 1.06, 95% CI, 1.01–1.11, *p = *0.01), LRNC presence (OR = 9.91, 95% CI, 2.66–36.88, *p = *0.001), and IPH presence (OR = 9.20, 95% CI, 2.32–36.39, *p *= 0.002) were correlated with recurrent stroke (Table 3).

**TABLE 3:** Univariate and multivariate logistic regression analyses of stroke recurrence.

Variable	Univariate analysis			Multivariate analysis		
	OR	95% CI	*p* value	OR	95% CI	*p* value
Sex, male, *n* (%)	1.43	0.34–5.91	0.620			
Age, years	1.01	0.95–1.08	0.628			
BMI, kg/m^2^	0.93	0.76–1.12	0.452			
Hypertension, *n* (%)	0.61	0.20–1.89	0.402			
Diabetes mellitus, *n* (%)	0.69	0.21–2.23	0.539			
Coronary heart disease, *n* (%)	0.68	0.07–6.00	0.732			
Hyperlipidemia, *n* (%)	1.70	0.46–6.20	0.417			
Prior stroke/TIA, *n* (%)	1.43	0.39–5.12	0.583			
Family history of stroke, *n* (%)	0.41	0.11–1.58	0.196			
Smoking history, *n* (%)	1.02	0.31–3.31	0.973			
Alcohol consumption, *n* (%)	0.97	0.31–2.95	0.959			
Hs‐CRP, mg/L	1.37	1.16–1.61	< 0.001	1.39	1.07–1.79	0.011
NLR	1.26	0.98–1.61	0.063			
HCY, µmol/L	1.01	0.98–1.04	0.302			
Triglycerides (mmol/L)	0.67	0.31–1.46	0.321			
Total cholesterol (mmol/L)	0.71	0.37–1.38	0.325			
High‐density lipoprotein cholesterol (mmol/L)	0.03	0.00–0.76	0.033	0.05	0–7.28	0.248
Low‐density lipoprotein cholesterol (mmol/L)	0.89	0.41–1.90	0.76			
Aspirin use until primary outcome, *n* (%)	1.08	0.34–3.35	0.892			
Statins use until primary outcome, *n* (%)	0.82	0.27–2.52	0.741			
Categories of stenotic degree	0.39	0.15–1.04	0.061			
NWI, %	1.06	1.01–1.12	0.001	1.08	1.01–1.15	0.032
Presence of plaque components, *n* (%)						
Calcification	1.26	0.38–4.13	0.701			
LRNC	9.91	2.66–36.88	0.001	9.01	1.13–71.47	0.037
IPH	9.20	2.32–36.39	0.002	8.12	1.06–62.13	0.043

Abbreviations: BMI, body mass index; HCY, homocysteine; Hs‐CRP, high‐sensitivity C‐reactive protein; IPH, intraplaque hemorrhage; LRNC, lipid‐rich necrotic core; NLR, neutrophil‐to‐lymphocyte ratio; NWI, normalized wall index; TIA, Transient ischemic attack.

Multivariate logistic regression analysis showed that Hs‐CRP (OR = 1.39, 95% CI, 1.07–1.79, *p = *0.011), NWI (OR = 1.08, 95% CI, 1.01–1.15, *p = *0.032), LRNC (OR = 9.01, 95% CI, 1.13–71.47, *p = *0.037), and IPH (OR = 8.12, 95% CI, 1.06–62.13, *p = *0.043) were associated with recurrent stroke (Table 3).

### 3.4 Predictors of Recurrent Stroke

**FIGURE 2:**
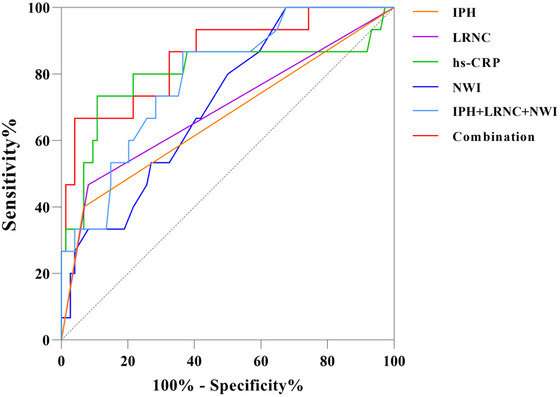
Receiver operating characteristic curves showing the diagnostic performances of serum Hs‐CRP, IPH, LRNC, and NWI levels for predicting stroke recurrence within 1 year. The combination of Hs‐CRP, IPH, LRNC, and NWI had a significantly higher AUC. Hs‐CRP, high‐sensitivity C‐reactive protein; IPH, intraplaque hemorrhage; LRNC, lipid‐rich necrotic core; NWI, normalized wall index.

The AUCs of NWI, Hs‐CRP, LRNC, IPH, and NWI+LRNC+IPH were 0.719 (95% CI, 0.614–0.809), 0.800 (95% CI, 0.701–0.877), 0.693 (95% CI, 0.586–0.786), 0.666 (95% CI, 0.558–0.763), and 0.787 (95% CI, 0.690–0.870), respectively. When Hs‐CRP was combined with NWI, LRNC, and IPH, the AUC increased to 0.855 (95% CI, 0.764–0.921). Delong's test showed that the combined model's diagnostic performance was superior to that of the models using NWI (*p *= 0.006), LRNC (*p *= 0.019), and IPH (*p *= 0.011) alone. The AUC of the combined model was slightly, but not significantly, higher than that of the Hs‐CRP model (*p *= 0.484). The ROC analyses are shown in Table 4 and Figure 2.

**TABLE 4:** Area under the curve values for selected predictors of stroke recurrence.

Variable	AUC	95%CI	Sensitivity	Specificity
NWI	0.719^a^	0.614–0.809	100.0	32.43
Hs‐CRP	0.800	0.701–0.877	73.33	89.19
LRNC	0.693^a^	0.586–0.786	46.67	91.89
IPH	0.666^a^	0.558–0.763	40.0	93.24
IPH+LRNC+NWI	0.787	0.690–0.870	86.67	63.51
Combination	0.855	0.764–0.921	66.67	95.95

Abbreviations: Combination = NWI+Hs‐CRP+LRNC+IPH; Hs‐CRP, high‐sensitivity C‐reactive protein; IPH, intraplaque hemorrhage; LRNC, lipid‐rich necrotic core; NWI, normalized wall index.

^a^ AUC compared with combination, *p *< 0.05.

### 3.5 Reproducibility

To assess the reproducibility, 20 patients were randomly selected from the study population. The ICC for the NWI was 0.872 (95% CI, 0.708–0.947). The Kappa values for the categories of stenotic degree, LRNC, calcification, and IPH were 0.828–1.000.

## 4 Discussion

We found that Hs‐CRP and plaque characteristics (i.e., NWI, LRNC, and IPH) were all independent risk factors for recurrent stroke. The model combining Hs‐CRP and carotid plaque characteristics achieved the highest diagnostic performance. Our findings may indicate that combining serum inflammatory markers and carotid plaque features will enable more effective decision‐making for clinically predicting recurrent ischemic events.

Hs‐CRP is one of the most commonly used serum biomarkers in clinical settings and is an important marker of the body's inflammatory condition during ischemic stroke (Lappegård et al. 2018). Our findings indicated that a higher serum Hs‐CRP level was an independent risk factor for stroke recurrence. A prior study also found that patients with recurrent stroke express high Hs‐CRP levels (R. Xu et al. 2019). In addition, aligning with our results, G. Wang et al. (2021) found that increased Hs‐CRP levels were correlated with about a 4.7‐fold increase in the risk of ischemic stroke within 1 year compared with that of non‐elevated Hs‐CRP levels in a randomized clinical trial with 807 patients. Although the combined model demonstrated a numerically higher AUC (0.855 vs. 0.800) compared to Hs‐CRP alone, the difference did not reach statistical significance. This suggests that while carotid plaque characteristics may provide incremental prognostic value, their clinical utility in combination with Hs‐CRP requires validation in larger cohorts. Future studies should incorporate decision‐curve analysis to evaluate whether this modest AUC improvement translates to tangible clinical benefit.

In our study, the NLR was higher in patients with recurrent stroke than in those without. A prior study reported that the NLR can be used to predict recurrent stroke (Ma et al. 2020). However, our results showed that the NLR was unassociated with recurrent stroke. A study on symptomatic intracranial arterial stenosis with stenting showed similar results (Haidegger et al. 2021). The reasons for the elevations of NLR are complex and may be related to the increase in neutrophil count and the decrease in lymphocyte count. Increased peripheral neutrophil levels are considered an indicator of the inflammatory response of endothelial cells (Pierini et al. 2019). Simultaneously, HCY levels were also found to be unassociated with recurrent stroke. Kumral et al. (2016) performed a study on 9522 participants with stroke and found that the increased HCY was not independently correlated with recurrent stroke or cardiovascular events. However, a prospective analysis of 1823 Chinese patients found that HCY was correlated with recurrent ischemic events (Zhang et al. 2009). There is still heterogeneity among the studies, and more research is still warranted.

We also observed that IPH was significantly correlated with recurrent stroke and had a high predictive performance for recurrent ischemic events. Che et al. (2022) followed 171 patients with acute ischemic events for a mean of 12 months and found that the presence of carotid IPH has a strong correlation with recurrent ischemic events and could be a predictor of stroke recurrence. The PARISK study also reported that IPH was an independent risk factor for the recurrence of stroke (van Dam‐Nolen et al. 2022). Furthermore, our results indicated that patients with LRNC presence had higher incidences of recurrent ipsilateral stroke. Previous studies have reported that LRNC and IPH are potential markers of plaque vulnerability (Saba et al. 2019). Vulnerable plaques are prone to triggering ischemic events as a result of cerebral emboli that arise from platelet‐rich thrombi on their surface or from the rupture of the plaque itself.

Patients with recurrent stroke tend to have greater plaque volume and plaque burden (Liu et al. 2012; van Dam‐Nolen et al. 2022). In this study, our results also demonstrates that NWI is an independent risk factor for the recurrence of stroke. However, there is no significant correlation between recurrent stroke and the degree of stenosis. These results indicate that NWI may have a significantly better predictive value for stroke recurrence than the traditional stenosis degree. This may be because the degree of stenosis is susceptible to interference from positive dilatation, and the true plaque burden was underestimated. In contrast, the NWI, a standardized indicator of plaque burden, is largely unaffected by individual differences in vessel size (Cao et al. 2017).

While our analysis focuses primarily on recurrent stroke events, it is of secondary but still notable interest to examine variables that might be associated with the absence of events, or “non‐events.” For instance, calcification in the plaque did not show a significant association with recurrent stroke in this study (OR = 1.26, 95% CI, 0.38–4.13, *p *= 0.701), but it might still offer insight into the protective mechanisms underlying the absence of stroke events. The potential for calcification to stabilize atherosclerotic plaques and reduce the risk of stroke recurrence warrants further investigation, particularly in larger cohorts or with advanced imaging modalities. Additionally, other variables such as lipid profile components, including HDL‐C, were analyzed but did not demonstrate a significant association with recurrent stroke in the multivariate analysis.

In this study, the combined model performed better than the other models in predicting recurrent stroke. These results confirmed the added value of combining serum inflammatory markers and plaque characteristics to predict recurrent stroke. Our study may provide a potential method for the early identification of high‐risk recurrent stroke patients, which will further contribute to the early prevention and treatment of recurrent stroke.

### 4.1 Limitations

This study still has several limitations. First of all, while the magnitude of LRNC's association with recurrence is notable, the small sample size underscores the need for cautious interpretation and further validation. Future multicenter studies with extended follow‐up periods are warranted to validate these preliminary results. Secondly, serum samples were only obtained on admission. A longitudinal analysis of the serum markers over several periods is warranted. Inflammation is a complex process, and the effects of various factors on inflammation after ischemic stroke remain unclear. Therefore, assessments involving only leukocytes and Hs‐CRP may be insufficient. Future studies should include more inflammatory markers to further validate our conclusions. Additionally, carotid plaque‐RADS (Saba et al. 2024) has been recently introduced; however, we did not analyze this score in our study. In future studies, we will analyze our data in combination with carotid plaque‐RADS to refine our results further. Lastly, the 1‐year follow‐up may be short when considering the period of recurrent strokes, which increases the possibility that some recurrent strokes may not have been captured within the 1‐year timeframe. Future studies with longer follow‐up periods are crucial to fully assess the prognostic value of the biomarkers and plaque characteristics identified in our study.

## 5 Conclusions

Serum Hs‐CRP level and plaque vulnerability characteristics are independent risk factors for recurrent stroke. The combination of serum inflammatory markers and plaque vulnerability characteristics may demonstrate better predictive potential for stroke recurrence risk, which may enable the precision identification of high‐risk populations and inform targeted prevention protocols.

## Author Contributions

**Lin Wang**: conceptualization, methodology, data curation, investigation, validation, writing – original draft, writing – review and editing. **Tong Chen**: methodology, software, validation. **Chun Yuan**: validation, investigation, formal analysis. **Thomas Hatsukami**: data curation, software, investigation, conceptualization, resources, project administration, writing – original draft. **Xihai Zhao**: validation, visualization, investigation. **Mi Shen**: validation, software, data curation. **Peiyi Gao**: data curation, software, supervision. **Xingquan Zhao**: writing – original draft, writing – review and editing, validation, conceptualization, project administration, methodology. **Binbin Sui**: conceptualization, methodology, validation, writing – original draft, writing – review and editing, project administration.

## Conflicts of Interest

The authors declare no conflicts of interest.

## Peer Review

The peer review history for this article is available at https://publons.com/publon/10.1002/brb3.70909.

## Supporting information




**Supplementary Material**: brb370909‐sup‐0001‐SuppMat.docx

## Data Availability

Data can be made available upon reasonable request.

## References

[brb370909-bib-0001] Cao, Y. , Y. Sun , B. Zhou , et al. 2017. “Atherosclerotic Plaque Burden of Middle Cerebral Artery and Extracranial Carotid Artery Characterized by MRI in Patients With Acute Ischemic Stroke in China: Association and Clinical Relevance.” Neurological Research 39, no. 4: 344–350. 10.1080/01616412.2017.1281196. 28136710

[brb370909-bib-0002] Che, F. , D. Mi , A. Wang , et al. 2022. “Extracranial Carotid Plaque Hemorrhage Predicts Ipsilateral Stroke Recurrence in Patients With Carotid Atherosclerosis—A Study Based on High‐Resolution Vessel Wall Imaging MRI.” BMC Neurology 22, no. 1: 237. 10.1186/s12883-022-02758-3. 35764942 PMC9238155

[brb370909-bib-0003] Cheng, S. F. , M. M. Brown , R. J. Simister , and T. Richards . 2019. “Contemporary Prevalence of Carotid Stenosis in Patients Presenting With Ischaemic Stroke.” British Journal of Surgery 106, no. 7: 872–878. 10.1002/bjs.11136. 30938840

[brb370909-bib-0004] DeLong, E. R. , D. M. DeLong , and D. L. Clarke‐Pearson . 1988. “Comparing the Areas Under Two or More Correlated Receiver Operating Characteristic Curves: A Nonparametric Approach.” Biometrics 44, no. 3: 837–845. 3203132

[brb370909-bib-0005] Ferguson, G. G. , M. Eliasziw , H. W. Barr , et al. 1999. “The North American Symptomatic Carotid Endarterectomy Trial : Surgical Results in 1415 Patients.” Stroke 30, no. 9: 1751–1758. 10.1161/01.str.30.9.1751. 10471419

[brb370909-bib-0006] GBD 2019 Diseases and Injuries Collaborators . 2020. “Global Burden of 369 Diseases and Injuries in 204 Countries and territories, 1990–2019: A Systematic Analysis for the Global Burden of Disease Study 2019.” Lancet 396, no. 10258: 1204–1222. 10.1016/S0140-6736(20)30925-9. 33069326 PMC7567026

[brb370909-bib-0007] Haidegger, M. , M. Kneihsl , K. Niederkorn , et al. 2021. “Blood Biomarkers of Progressive Atherosclerosis and Restenosis After Stenting of Symptomatic Intracranial Artery Stenosis.” Scientific Reports 11, no. 1: 15599. 10.1038/s41598-021-95135-y. 34341413 PMC8329296

[brb370909-bib-0008] Kumral, E. , G. Saruhan , D. Aktert , and M. Orman . 2016. “Association of Hyperhomocysteinemia With Stroke Recurrence After Initial Stroke.” Journal of Stroke and Cerebrovascular Diseases 25, no. 8: 2047–2054. 10.1016/j.jstrokecerebrovasdis.2016.05.008. 27260368

[brb370909-bib-0009] Kwee, R. M. , R. J. van Oostenbrugge , W. H. Mess , et al. 2013. “MRI of Carotid Atherosclerosis to Identify TIA and Stroke Patients Who Are at Risk of a Recurrence.” Journal of Magnetic Resonance Imaging 37, no. 5: 1189–1194. 10.1002/jmri.23918. 23166040

[brb370909-bib-0010] Lappegård, J. , T. S. Ellingsen , K. Hindberg , et al. 2018. “Impact of Chronic Inflammation, Assessed by hs‐CRP, on the Association Between Red Cell Distribution Width and Arterial Cardiovascular Disease: The Tromsø Study.” TH Open 2, no. 2: e182–e189. 10.1055/s-0038-1651523. 31249941 PMC6524874

[brb370909-bib-0011] Li, J. , Y. Pan , J. Xu , et al. 2021. “Residual Inflammatory Risk Predicts Poor Prognosis in Acute Ischemic Stroke or Transient Ischemic Attack Patients.” Stroke 52, no. 9: 2827–2836. 10.1161/STROKEAHA.120.033152. 34281380

[brb370909-bib-0012] Li, J. , X. Zhao , X. Meng , et al. 2016. “High‐Sensitive C‐Reactive Protein Predicts Recurrent Stroke and Poor Functional Outcome: Subanalysis of the Clopidogrel in High‐Risk Patients With Acute Nondisabling Cerebrovascular Events Trial.” Stroke 47, no. 8: 2025–2030. 10.1161/STROKEAHA.116.012901. 27328699

[brb370909-bib-0013] Li, J. , D. Li , D. Yang , et al. 2020. “Irregularity of Carotid Plaque Surface Predicts Subsequent Vascular Event: A MRI Study.” Journal of Magnetic Resonance Imaging 52, no. 1: 185–194. 10.1002/jmri.27038. 31944452

[brb370909-bib-0014] Liu, X. S. , H. L. Zhao , Y. Cao , Q. Lu , and J. R. Xu . 2012. “Comparison of Carotid Atherosclerotic Plaque Characteristics by High‐Resolution Black‐Blood MR Imaging Between Patients With First‐Time and Recurrent Acute Ischemic Stroke.” American Journal of Neuroradiology 33, no. 7: 1257–1261. 10.3174/ajnr.A2965. 22345496 PMC7965510

[brb370909-bib-0015] Lozano, R. , M. Naghavi , K. Foreman , et al. 2012. “Global and Regional Mortality From 235 Causes of Death for 20 Age Groups in 1990 and 2010: A Systematic Analysis for the Global Burden of Disease Study 2010.” Lancet 380, no. 9859: 2095–2128. 10.1016/S0140-6736(12)61728-0. 23245604 PMC10790329

[brb370909-bib-0016] Lu, M. , P. Peng , Y. Cui , et al. 2018. “Association of Progression of Carotid Artery Wall Volume and Recurrent Transient Ischemic Attack or Stroke: A Magnetic Resonance Imaging Study.” Stroke 49, no. 3: 614–620. 10.1161/STROKEAHA.117.019422. 29382804

[brb370909-bib-0017] Ma, Z. , Y. Yue , Y. Luo , W. Wang , Y. Cao , and Q. Fang . 2020. “Clinical Utility of the Inflammatory Factors Combined With Lipid Markers in the Diagnostic and Prognostic Assessment of Ischemic Stroke: Based on Logistic Regression Models.” Journal of Stroke and Cerebrovascular Diseases 29, no. 4: 104653. 10.1016/j.jstrokecerebrovasdis.2020.104653. 32033900

[brb370909-bib-0018] Mandell, D. M. , M. Mossa‐Basha , Y. Qiao , et al. 2017. “Intracranial Vessel Wall MRI: Principles and Expert Consensus Recommendations of the American Society of Neuroradiology.” American Journal of Neuroradiology 38, no. 2: 218–229. 10.3174/ajnr.A4893. 27469212 PMC7963837

[brb370909-bib-0019] Marnane, M. , S. Prendeville , C. McDonnell , et al. 2014. “Plaque Inflammation and Unstable Morphology Are Associated With Early Stroke Recurrence in Symptomatic Carotid Stenosis.” Stroke 45, no. 3: 801–806. 10.1161/STROKEAHA.113.003657. 24481971

[brb370909-bib-0020] Martinez, E. , J. Martorell , and V. Riambau . 2020. “Review of Serum Biomarkers in Carotid Atherosclerosis.” Journal of Vascular Surgery 71, no. 1: 329–341. 10.1016/j.jvs.2019.04.488. 31327598

[brb370909-bib-0021] Ojima, S. , T. Kubozono , S. Kawasoe , et al. 2020. “Association of Risk Factors for Atherosclerosis, Including High‐Sensitivity C‐Reactive Protein, With Carotid Intima‐Media Thickness, Plaque Score, and Pulse Wave Velocity in a Male Population.” Hypertension Research 43, no. 5: 422–430. 10.1038/s41440-019-0388-2. 31980747

[brb370909-bib-0022] Pakizer, D. , J. Kozel , J. Elmers , et al. 2025. “Diagnostics Accuracy of Magnetic Resonance Imaging in Detection of Atherosclerotic Plaque Characteristics in Carotid Arteries Compared to Histology: A Systematic Review.” Journal of Magnetic Resonance Imaging 61, no. 3: 1067–1093. 10.1002/jmri.29522. 38981139 PMC11803704

[brb370909-bib-0023] Pierini, A. , E. Gori , I. Lippi , G. Ceccherini , G. Lubas , and V. Marchetti . 2019. “Neutrophil‐to‐Lymphocyte Ratio, Nucleated Red Blood Cells and Erythrocyte Abnormalities in Canine Systemic Inflammatory Response Syndrome.” Research in Veterinary Science 126: 150–154. 10.1016/j.rvsc.2019.08.028. 31493682

[brb370909-bib-0024] Saba, L. , R. Cau , A. Murgia , et al. 2024. “Carotid Plaque‐RADS: A Novel Stroke Risk Classification System.” JACC: Cardiovascular Imaging 17, no. 1: 62–75. 10.1016/j.jcmg.2023.09.005. 37823860

[brb370909-bib-0025] Saba, L. , T. Saam , H. R. Jäger , et al. 2019. “Imaging Biomarkers of Vulnerable Carotid Plaques for Stroke Risk Prediction and Their Potential Clinical Implications.” Lancet Neurology 18, no. 6: 559–572. 10.1016/S1474-4422(19)30035-3. 30954372

[brb370909-bib-0026] van Dam‐Nolen, D. H. K. , M. T. B. Truijman , A. G. van der Kolk , et al. 2022. “Carotid Plaque Characteristics Predict Recurrent Ischemic Stroke and TIA: The PARISK (Plaque At RISK) Study.” JACC: Cardiovascular Imaging 15, no. 10: 1715–1726. 10.1016/j.jcmg.2022.04.003. 36202450

[brb370909-bib-0027] Wang, G. , J. Jing , J. Li , et al. 2021. “Association of Elevated hs‐CRP and Multiple Infarctions With Outcomes of Minor Stroke or TIA: Subgroup Analysis of CHANCE Randomised Clinical Trial.” Stroke and Vascular Neurology 6, no. 1: 80–86. 10.1136/svn-2020-000369. 32958697 PMC8005909

[brb370909-bib-0028] Wang, W. , B. Jiang , H. Sun , et al. 2017. “Prevalence, Incidence, and Mortality of Stroke in China: Results From a Nationwide Population‐Based Survey of 480 687 Adults.” Circulation 135, no. 8: 759–771. 10.1161/CIRCULATIONAHA.116.025250. 28052979

[brb370909-bib-0029] Han, J. , M. Wang , J. Jing , et al. 2024. “Trends and Risk Factors Associated With Stroke Recurrence in China, 2007–2018.” Stroke 52, no. 9: 2827–2836. 10.1001/jamanetworkopen.2022.16341.

[brb370909-bib-0030] Xu, R. , Y. Zhang , X. Gao , Y. Wan , and Z. Fan . 2019. “High‐Sensitivity CRP (C‐Reactive Protein) Is Associated With Incident Carotid Artery Plaque in Chinese Aged Adults.” Stroke 50, no. 7: 1655–1660. 10.1161/STROKEAHA.119.025101. 31195938

[brb370909-bib-0031] Zhang, W. , K. Sun , J. Chen , et al. 2009. “High Plasma Homocysteine Levels Contribute to the Risk of Stroke Recurrence and All‐Cause Mortality in a Large Prospective Stroke Population.” Clinical Science 118, no. 3: 187–194. 10.1042/CS20090142. 19515015

[brb370909-bib-0032] Zhao, X. , D. S. Hippe , R. Li , et al. 2017. “Prevalence and Characteristics of Carotid Artery High‐Risk Atherosclerotic Plaques in Chinese Patients With Cerebrovascular Symptoms: A Chinese Atherosclerosis Risk Evaluation II Study.” Journal of the American Heart Association 6, no. 8: e005831. 10.1161/JAHA.117.005831. 28862936 PMC5586432

[brb370909-bib-0033] Zhou, Z. , R. Li , X. Zhao , et al. 2015. “Evaluation of 3D Multi‐Contrast Joint Intra‐ and Extracranial Vessel Wall Cardiovascular Magnetic Resonance.” Journal of Cardiovascular Magnetic Resonance 17, no. 1: 41. 10.1186/s12968-015-0143-z. 26013973 PMC4446075

