# Supplementary material for: Carotid Plaque Characteristics Combined With Serum Inflammatory Biomarkers Predict Recurrent Ischemic Stroke
Source: Brain Behav. 2025 Oct 7;15(10):e70909. doi: 10.1002/brb3.70909 (PMC12504801; doi:10.1002/brb3.70909)
Supplement: Supplementary file 1 — Supplementary Material: brb370909‐sup‐0001‐SuppMat.docx [file BRB3-15-e70909-s001.docx]

**Supplementary information**

Table S1. Magnetic resonance sequences and parameters for carotid VWI

|  | TOF | 3D-T1 SPACE | MP-RAGE |
| --- | --- | --- | --- |
| TR, ms | 21 | 800 | 13 |
| TE, ms | 5 | 22 | 4 |
| FOV, cm | 27×40 | 18×18 | 14×14 |
| Resolution, mm^2^ | 0.6×0.6 | 0.8×0.8 | 0.8×0.8 |
| Slice thickness, mm | 1.0 | 0.8 | 0.8 |
| Fat suppression | No | Yes | Yes |

Abbreviations: VWI, vessel wall imaging; TOF, time-of-flight; MP-RAGE, magnetization-prepared rapid-acquisition gradient echo; TR, repetition time; TE, echo time; FOV, field of view; SPACE, sampling perfection with application of optimized contrasts using different flip angle evolutions


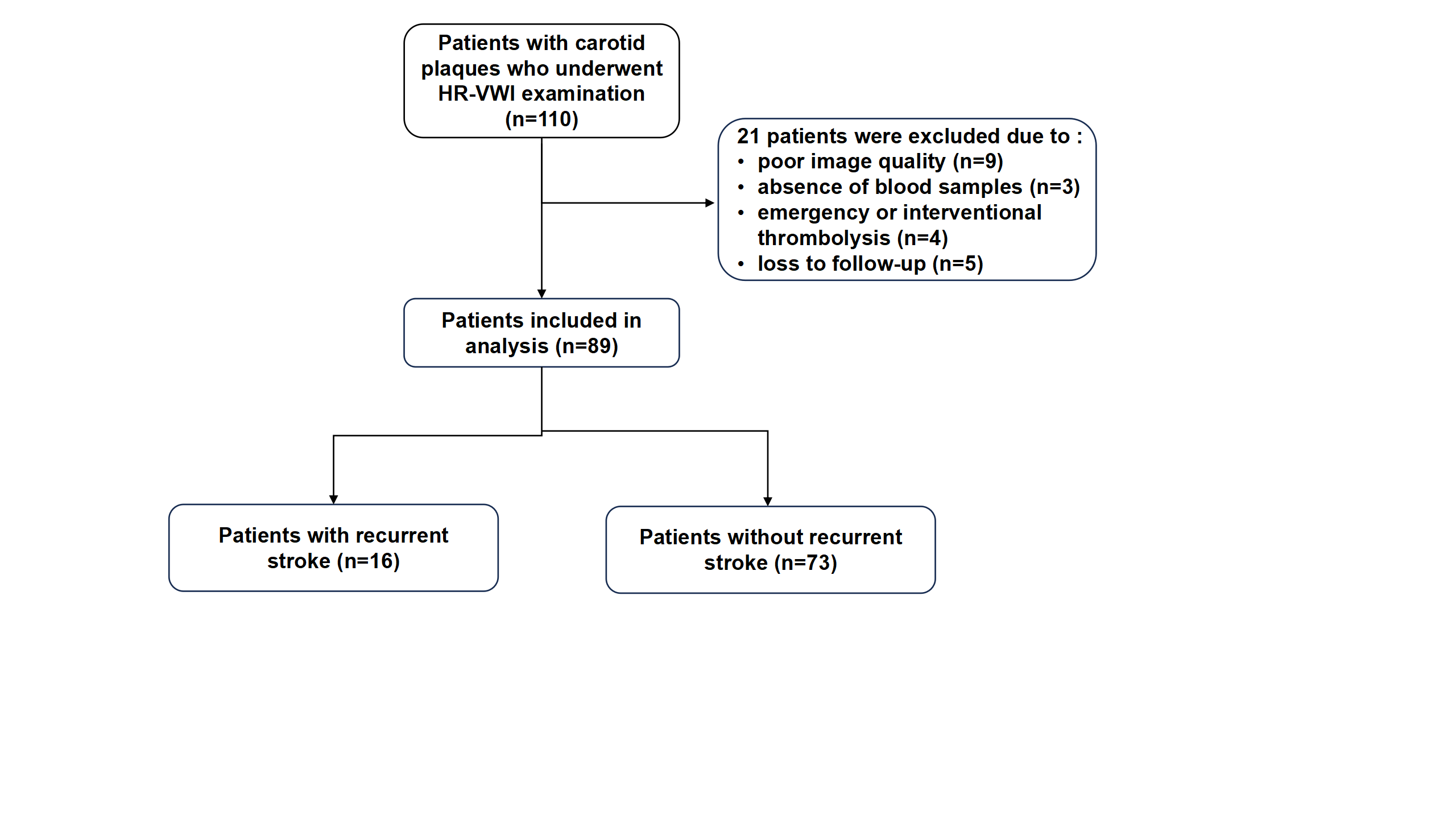


Figure S1. Flowchart of patient inclusion. In total, 110 patients with cerebral infarction underwent carotid VWI; 21 were excluded for the following: poor image quality (n=9), absence of 24-h blood samples (n=3), emergency or interventional thrombolysis (n=4), and loss to follow-up (n=5). Finally, 89 patients were included in the analysis.
